# Seed Dispersal and Germination Traits of 70 Plant Species Inhabiting the Gurbantunggut Desert in Northwest China

**DOI:** 10.1155/2014/346405

**Published:** 2014-11-17

**Authors:** Huiliang Liu, Daoyuan Zhang, Xuejun Yang, Zhenying Huang, Shimin Duan, Xiyong Wang

**Affiliations:** ^1^Key Laboratory of Biogeography and Bioresource in Arid Land, Xinjiang Institute of Ecology and Geography, Chinese Academy of Sciences, Urumqi 830011, China; ^2^Turpan Eremophytes Botanical Garden, Chinese Academy of Sciences, Turpan 838008, China; ^3^State Key Laboratory of Vegetation and Environmental Change, Institute of Botany, Chinese Academy of Sciences, Beijing 100093, China

## Abstract

Seed dispersal and germination were examined for 70 species from the cold Gurbantunggut Desert in northwest China. Mean and range (3 orders of magnitude) of seed mass were smaller and narrower than those in other floras (5–8 orders of magnitude), which implies that selection favors relatively smaller seeds in this desert. We identified five dispersal syndromes (anemochory, zoochory, autochory, barochory, and ombrohydrochory), and anemochorous species were most abundant. Seed mass (*F* = 3.50, *P* = 0.01), seed size (*F* = 8.31, *P* < 0.01), and seed shape (*F* = 2.62, *P* = 0.04) differed significantly among the five dispersal syndromes and barochorous species were significantly smaller and rounder than the others. There were no significant correlations between seed mass (seed weight) (*P* = 0.15), seed size (*P* = 0.38), or seed shape (variance) (*P* = 0.95) and germination percentage. However, germination percentages differed significantly among the dispersal syndromes (*F* = 3.64, *P* = 0.01) and seeds of ombrohydrochorous species had higher germination percentages than those of the other species. In the Gurbantunggut Desert, the percentage of species with seed dormancy was about 80%. In general, our studies suggest that adaptive strategies in seed dispersal and germination of plants in this area are closely related to the environment in which they live and that they are influenced by natural selection forces.

## 1. Introduction

Each desert plant species has its own complex life history strategy that enables it to persist in its arid habitat. These strategies include seed dispersal and germination [1]. In general, seed dispersal helps seeds escape sibling and kin competition, decreases predation, reduces the probability of offspring survival in unpredictable environments, and aids in reaching and colonizing new habitats for seed germination and population regeneration [2–6]. Different plants evolve different dispersal strategies in deserts. The escape strategy occurs when plants produce large numbers of small seeds with long viability and thus escape from seed-eating insects and other animals by entering cracks in the soil. The protection strategy occurs when mature seeds remain attached to the mother plant and must be dispersed by wind, animals, or rain and it reduces the potential of dispersal to uncertain conditions [7]. In addition, seed structures and characters (seed mass and seed shape) affect the effectiveness of seed dispersal [8, 9]. Hence, species with different structures form different dispersal syndromes, including anemochory [10–12], zoochory [13–23], autochory [24, 25], ombrohydrochory [7, 25], and barochory [26]. However, very little is known about the dispersal strategy in relation to dispersal syndromes in plant species of cold deserts.

Seed germination and dormancy are pivotal events in seedling establishment, and they are closely related to seed dispersal and population generation. Some researchers consider seed dormancy as an adaptive bet-hedging strategy in spatiotemporally varying environments [27, 28]. In other words, desert plants depend more on dormancy than dispersal for survival, since timing of precipitation is uncertain and plants tend to delay germination until the occurrence of a favorable precipitation event rather than relying on dispersal [24, 29]. For this reason, seed dormancy and germination are important for desert plants. Species inhabiting deserts have developed different seed germination straregies via natural selection over a long period or time [1]. Typically, these strategies are divided into two general categories: (1) opportunistic, that is, with fast germination rate, no or low seed dormancy, and high germination percentage; and (2) cautious germination strategies, with low germination rate, high seed dormancy, and low germination percentage [1, 7]. In addition, seed dispersal strategies seem to be related with germination strategies. Plants in the Negev Desert with seed protection strategies may develop cautious germination strategies with low risk to seedling survival, while plants with escape seed dispersal strategies may develop opportunistic germination strategies with high risk to seedling survival [7]. However, whether those results apply to other desert regions is still unknown.

The Gurbantunggut Desert is located in the center of the Junggar Basin, Xinjiang Province, China, and it is the second largest desert in China with an area of 48,800 km^2^. In this area, there are 208 species of seed plants that belong to 30 families and 123 genera. Dominant families are Amaranthaceae, Asteraceae, Brassicaceae, Fabaceae, Poaceae, Polygonaceae, Tamaricaceae, and Zygophyllaceae and they contain 74% of all species in the Gurbantunggut Desert [30]. Hence, seed characteristics of species in this desert are diverse and complex [30]. Previous studies showed that diaspore mass and shape differed significantly among phylogenetic groups and dispersal syndromes in this desert [31]. However, germination strategies as well as seed dispersal strategies of species are adaptations to the desert environment, but very little is known about the relationship of seed dispersal and germination strategies in deserts (but see [7]). Our primary aim was to determine the relationships between seed dispersal and germination strategies in the Gurbantunggut Desert.

Information on seed dispersal and seed germination of desert plants is crucial to understanding adaptative strategies of plants in these arid areas. For the Gurbantunggut Desert, we asked the following questions. (1) Do escape and protection dispersal strategies exist? If they do, what is the proportion of each dispersal strategy? (2) Are seed traits related to seed dispersal sydromes, that is, what is the relationship between dispersal strategies and dispersal syndromes? (3) Do cautious and opportunistic germination strategies exsit? If they do, what is the proportion of each germination strategy, that is, what is the relationship between dispersal strategies and germination strategies? To answer these questions, we observed and measured seed structure, seed mass, seed size, seed shape to determine dispersal strategies, and syndromes and conducted seed germination experiments.

## 2. Materials and Methods

### 2.1. Study Site and Species

Because of the rain shadow from the Himalayan and Tian Shan ranges, moist air currents from the Indian Ocean do not reach the Gurbantunggut, resulting in a vast arid expanse. Mean annual precipitation is approximately 80 mm, falling predominantly during spring. Mean potential annual evaporation is >2600 mm. The mean annual temperature is 7.3°C. Wind speeds are highest in late spring (mean ≤ 11 m s^−1^), and wind is predominantly from the WNW, NW, and N [32]. However, compared with the Taklamakan Desert, there are more species in the Gurbantunggut Desert [30]. In 2007, we collected seeds of 70 species, that is, >33% of the total flora. For each species, their family, life form, and month of seed collecting were recorded (Table 1). The plants were divided into two types: (1) long-lived (L) (annuals (LA), perennial (LP), and shrubs (LS)) and short-lived or ephemeral (E) [33].

### 2.2. Seed Collection

Freshly matured intact natural dispersal units [34] of 70 species (Table 1) were collected at the time of seed dispersal from wild populations in the Gurbantunggut Desert in 2007. Seeds of each species were collected from more than 20 haphazardly chosen individuals, allowed to dry at room temperatures and then stored dry in paper bags (20°C; RH: 20–30%) until measurements were begun.

### 2.3. Seed Dispersal Traits and Strategies

For each species, seeds traits and dispersal syndromes and strategies were determined and classified by follows.

*Seed Mass*. Mean seed mass was determined by weighing three replicates of 100 intact seeds. Seeds with signs of pathogen or insect damage were not included. For statistical analyses, seed mass was log_10_ transformed to logarithms to stabilize variance and improve the distribution of residuals.

*Seed Size*. Seed diameter, defined as the largest diameter along the longest axis of a seed, was used as an index of seed size. Seed diameter was treated as a continuous variable for some exploratory analyses of the variance of seed diameter within the seed dispersal strategy. In such cases, seed diameter was log⁡_*e*_ transformed to stabilize variance.

*Seed Shape*. According to Thompson et al.'s methods [35], seed shape was calculated as the variance of the three main perpendicular dimensions of the seed after dividing all values by length.

*Dispersal Syndromes*. We assigned each of the 70 study species to one of five dispersal syndromes: zoochory, anemochory, autochory, barochory, and ombrohydrochory (Table 2) [32].

*Dispersal Strategies*. Following Gutterman's categories of dispersal strategies and our personal observations in the field, we recognized three dispersal strategies [7]: (1) species with seed mass < 1 mg and no aerial seed bank as an escape strategy, (2) species with aerial seed bank and reliance of seed dispersal on external elements as a protection strategy, and (3) escape-protection strategy. Although these categories are artificial, they can represent some realistic cases.

### 2.4. Seed Germination Traits

Germination percentages of fresh seeds of the 70 species were determined under laboratory conditions. For each species, four replicates of 50 seeds were incubated on two layers of moist filter paper in 9 cm diameter Petri dishes at daily (12 h/12 h) light (fluorescent light, 30 *μ*mol m^−2^ s^−1^) and dark period at 25/10°C (12 h/12 h). This temperature regime simulates the mean high and mean low temperatures in the field during the spring germination period. Germination was monitored every 24 h for 30 days and a seed was considered to be germinated if the radicle was visible to the naked eye; germinated seeds were counted and then removed. Final percentage germination (FPG) was calculated as FPG = GN/SN, where FPG is the final germination percentage, GN is the total number of germinated seeds, and SN is the total number of viable seeds. Final germination percentage was arcsine transformed as needed for statistical analyses.

### 2.5. Data Analyses

Data were analyzed using SPSS 15.0 (SPSS, Chicago, USA). One-way ANOVA was used to test for differences (*P* ≤ 0.05) in seed mass, seed size, seed shape and germination, dispersal strategies, and syndromes. Tukey's multiple comparison test was used to test for differences among seed dispersal syndromes, dispersal strategies, mass, size, shape, and germination percentages. The relationships among variables (e.g., seed mass and germination, seed size and germination, and seed shape and germination) also were analyzed by linear regression. Seed mass was log_10_-transformed, seed size (diameter) log⁡_*e*_-transformed, and seed germination percentage arcsin-transformed before analyses.

## 3. Results

### 3.1. Species and Seed Traits

**Figure 1:**
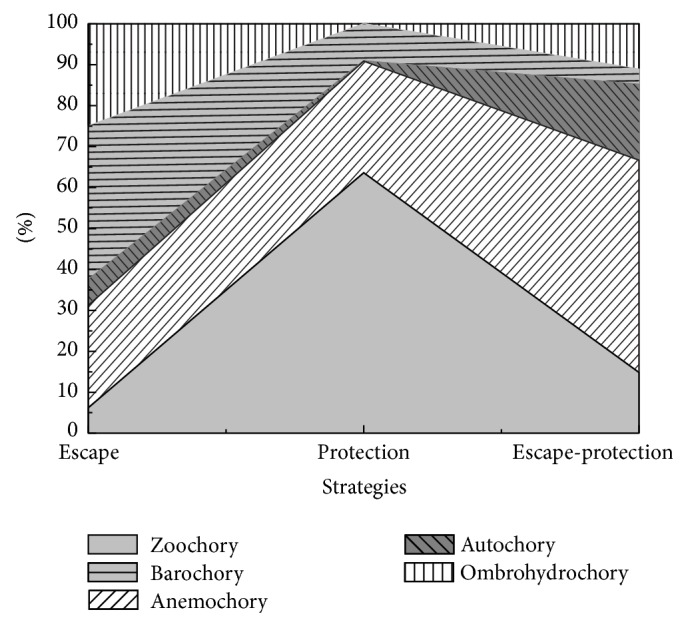
Proportion of dispersal syndromes in the three different dispersal strategies of species in the Gurbantunggut Desert.

**Figure 2:**
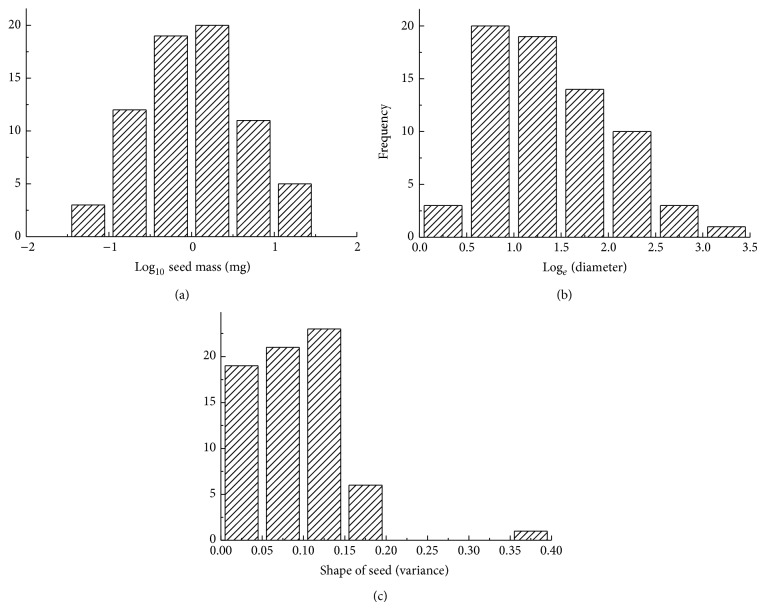
Frequency distribution of seed mass (a), seed size (b), and seed shape (c) of the species in the Gurbantunggut Desert.

The 70 species belong to 15 families and 48 genera, which accounted for 33.7% of the species, 39.0% of the genera, and 50% of the families in the Gurbantunggut Desert. Amaranthaceae was the most common family and included 18 genera and 32 species (Table 1). The proportion of dispersal syndromes was 17.2%, 35.7%, 10%, 21.4%, and 15.7% for zoochory, anemochory, autochory, barochory, and ombrohydrochory, respectively. The number of species with escape strategies, protection strategies, and escape-protection strategies was 32, 11, and 27, respectively. In the escape strategies, the dispersal syndrome with the highest frequency was barochory and that with the lowest frequency was zoochory. Yet, zoochory was highest syndrome of the protection strategies. In the escape-protection strategies, barochory was the least and anemochory the most frequent (Figure 1). Mean mass of 100 seeds ranged from 0.054 to 106.53 mg, with a mean and median for all 70 species of 5.16 and 1.02 mg, respectively (Table 1). The frequency of seed mass had a log-normal distribution that extended across three orders of magnitude (Figure 2(a)); thus, there were many more small-seeded than large-seeded species. Seed diameter ranged from 0.50 to 28.59 mm, with a mean and median of 4.06 and 2.63 mm, respectively (Table 1). The frequency of seed size also was skewed towards smaller seeds (Figure 2(b)). Seed shape ranged from 0.03 to 0.36, with a mean and median of 0.092 and 0.086, respectively (Table 1), indicating that a wide range of seed shapes was present. The frequency of seed shape (variance) also was skewed towards round (Figure 2(c)).

### 3.2. Relationship of Seed Traits with Dispersal Syndromes and Dispersal Strategies

**Figure 3:**
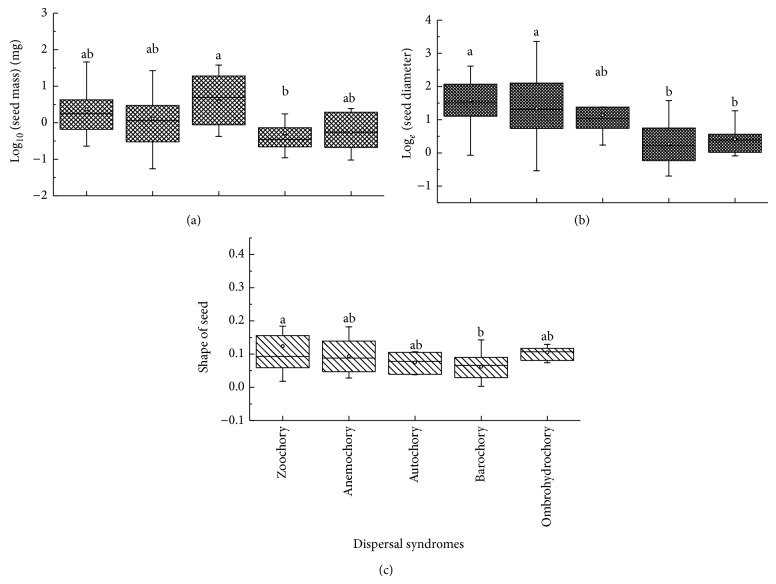
Box plots showing mean (∘), median (—), quartiles, and outliers (-) of seed mass (a), seed size (b), and seed shape (c) of 70 species grouped by dispersal syndromes. Different letters indicate subsets with significant difference (Tukey's test, *P* < 0.05).

The range of seed mass and seed size for dispersal syndromes was greatest in anemochorous species, whereas seed mass was greatest in autochorous species and least in barochorous species. Seed size was largest for zoochorous species and smallest for barochorous species. Zoochorous species had the widest range and largest variance for seed shape and barochorous species the smallest. Seed mass (*F* = 3.50, *P* = 0.01), seed size (*F* = 8.31, *P* < 0.01), and seed shape (*F* = 2.62, *P* = 0.04) differed significantly among the five dispersal syndromes, with barochorous species being significantly slighter, smaller, and rounder than the others (Figure 3).

**Figure 4:**
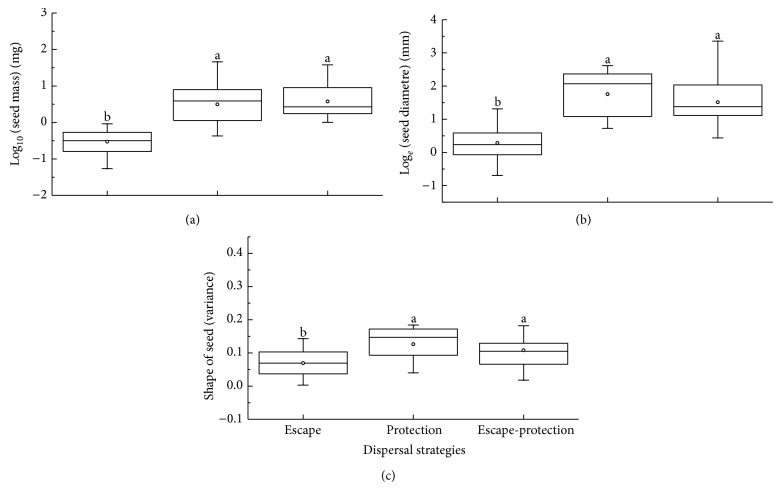
Box plots showing mean (∘), median (—), quartiles, and outliers (-) of seed mass (a), seed size (b), and seed shape (c) of 70 species grouped by dispersal strategies. Different letters indicate subsets with significant difference (Tukey's test, *P* < 0.05).

Species with the escape-protection strategy had the largest and widest range of seed mass and those with the escape strategy the narrowest range and lowest seed mass. Species with the escape-protection strategy had the widest range of seed size and of shape variance. Species with the protection strategy had the largest seed size and most irregular seed shape and those with the escape strategy the smallest seed size and roundest seed shape. Seed mass (*F* = 48.94, *P* < 0.01), seed size (*F* = 38.54, *P* < 0.01), and seed shape (*F* = 6.98, *P* < 0.01) differed significantly among the three dispersal strategies, and seed mass of species with the escape strategy was significantly smaller and rounder than that of other species (Figure 4).

### 3.3. Relationship of Seed Traits, Dispersal Syndromes, and Dispersal Strategies with Seed Germination

**Figure 5:**
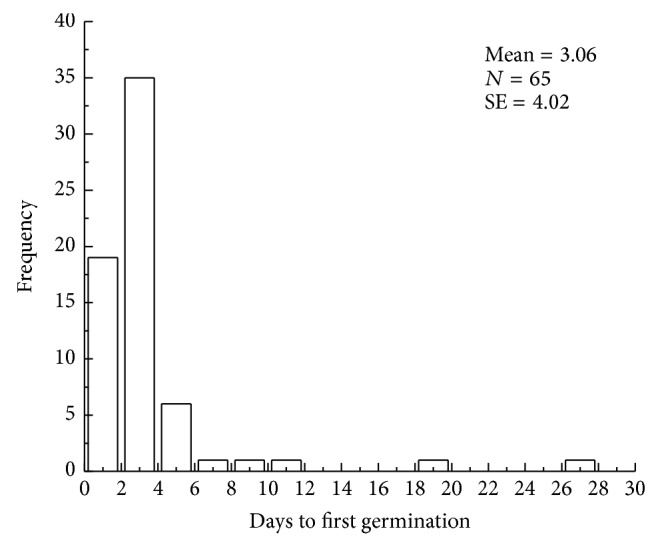
Frequency distribution of days to first germination of the species.

Days to first germination (DFG) ranged from 1 day (i.e.,* Plantago maritime subsp. ciliata, Haloxylon ammodendron, H. persicum, Bassia dasyphylla, B. hyssopifolia, Camphorosma monspeliaca, Anabasis aphylla, Kochia iranica, Salsola heptapotamica, S. nitraria, S. ruthenica, Suaeda acuminata, S. altissima, S. physophora, Petrosimonia sibirica, Halogeton glomeratus, Tamarix hispida, Trigonella arcuata, *and* T. cancellata*) to 27 days (*Leymus angustus*) and were skewed toward short periods of time (Figure 5). Final germination percentage ranged from 0 to 99%, with a mean and median of 42 and 31%, respectively (Table 1). Except for* Trigonella arcuata* and* T. cancellata*, seeds of Fabaceae (six species) had physical dormancy (PY), with germination percentages less than 10%. Physiological dormancy (PD) was the most common kind of dormancy (74.3%), and two species had either morphological dormancy (MD) or morphophysiological dormancy (MPD). The proportion of species that produced dormant seeds was 82.9% (see [53] for criteria to use in determining kinds of seed dormancy).

**Figure 6:**
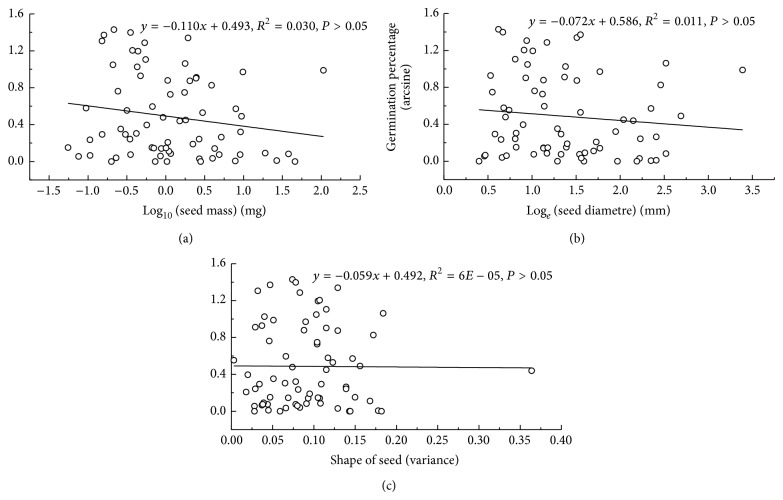
Relationships between mean seed mass (log_10_) (a), seed size (log⁡_*e*_⁡) (b), and seed shape (c), and mean arcsine square root final germination percentage of 70 species in the Gurbantunggut Desert.

**Figure 7:**
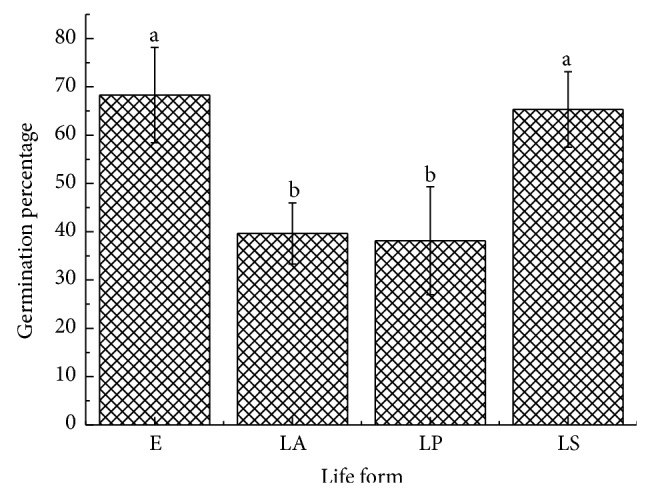
Mean germination percentage of four plant life forms. Biennials (<3 species) were excluded from the data.

**Figure 8:**
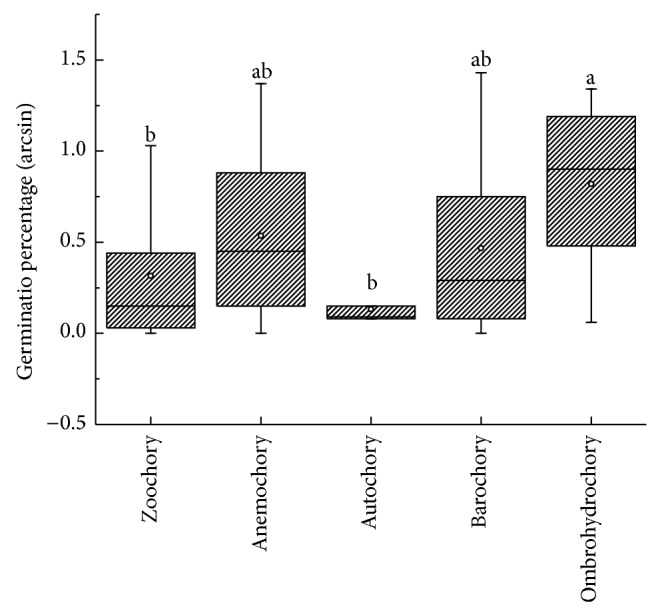
Box plots showing mean (∘), median (—), quartiles, and outliers (-) of seed germination percentages (arcsin) of 70 species grouped by dispersal syndromes. Difference letters indicate subsets with significant difference (Tukey's test, *P* < 0.05).

**Figure 9:**
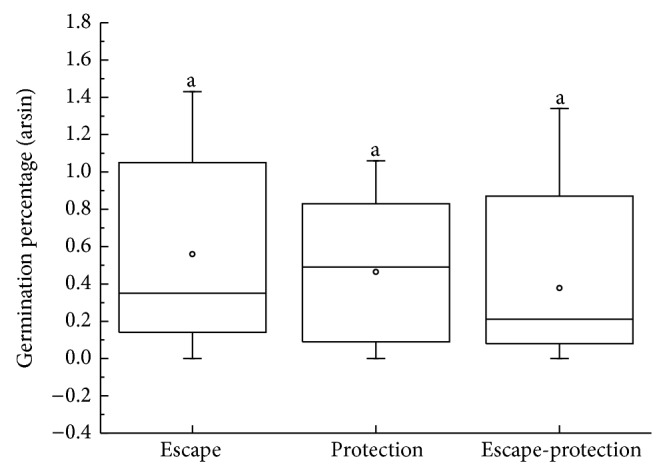
Box plots showing mean (∘), median (—), quartiles, and outliers (-) of seed germination percentages (arcsin) of 70 species grouped by dispersal strategies. Difference letters indicate subsets with significant difference (Tukey's test, *P* < 0.05).

Correlations between seed mass (*P* = 0.15, Figure 6(a)), seed size (*P* = 0.38, Figure 6(b)), seed shape (variance) (*P* = 0.95, Figure 6(c)), and germination percentages were positive but not significant. The rank-order of germination was EP > LSP > LAP > LPP (Figure 7). Germination percentages differed significantly among dispersal syndromes (*F* = 3.64, *P* = 0.01), with seeds of ombrohydrochorous species being the highest (Figure 8). Germination percentages did not differ significantly among dispersal strategies (*F* = 1.23, *P* = 0.30; Figure 9).

## 4. Discussion

### 4.1. Species and Seed Traits

Seed mass and shape are likely to be pivotal ecological traits for seedling establishment, formation of a persistent seed bank, and dispersal. Seed mass via the quantity of stored food reserves also affects plant regeneration, vegetative growth, and survival [35–39]. Generally, large seeds increase the chance of seedling survival and establishment [39, 40], while small seeds contribute more to forming seed bank [35] and to long distance dispersal [24]. In the Gurbantunggut Desert, mean and range of seed mass of 70 species (Table 1, Figure 2(a)) were much smaller and narrower compared with the other temperate floras [26, 41, 42]. This result is consistent with the conclusion that selection favors small seeds in desert species [43, 44].

Our study showed that seed shape tended to be round or ellipse, which favours formation of a persistent soil seed bank, while larger, flattened, or elongate seeds are likely to form a transient soil seed bank [35]. The approximately log-normal distribution of seed mass means that there are many more small-seeded than large-seeded species in the Gurbantunggut Desert. Why might this be? Shortage of resources for plants in deserts often constrains reproductive effort [1]. Evolution of plants in the Gurbantunggut Desert as well as herbaceous angiosperm in other ecosystems has resulted in them producing small and round seeds that maximize the number of seeds produced in an environment, when plants have limited ability to allocate resources to reproduction [45].

### 4.2. Strategies of Seed Dispersal

Results of our study also show correlations between seed mass and seed dispersal syndromes. Barochorous seeds tended to be smaller and more rounded than the others (Figure 2), whereas seeds of species with other dispersal syndromes tended to be big and flat, which is consistent with findings in the other geographic areas [26, 44, 46–48]. When seeds ripen, small seeded plants quickly disperse their seeds and they are more easily buried and more likely to fall into cracks in the soil or be washed in by rainwater than large seeds [49], which helps small seeds form a persistent or transient seed bank and thus to escape predators [35].

In other temperate areas, barochorous species are significantly more frequent than anemochorous and zoochorous species [26, 44, 50], but anemochorous species had the highest proportion of species in our study area (Figure 1). We considered that seed having wings, hairs, and balloons are dispersed far distances by wind in the Gurbantunggut Desert, which may contribute to plant colonization of a new sites [51].

Because of a low amount of food in many deserts, massive numbers of seeds may be consumed by local animals, especially ants, the main seed predators in desert areas [7]. In our study area, seeds of about 70% of the species were dispersed by ants, which suggest that most seeds are eaten by ants [31]. Two possible advantage of small seeds in deserts are (1) reduction of risk of being eaten by predators [52] and (2) falling into soil cracks and avoid detection by predators [7]. Thus, small seeds could form a long-lived seed bank in the Gurbantunggut Desert, which ensures survival of the species under predation stress as well as under extreme desert conditions.

### 4.3. Strategies of Seed Germination

Seed germination traits are linked to water availability, biogeography, seed mass, seed shape, and plant life form. In deserts, the water necessary for seedling establishment is available following precipitation and snowmelt and thus seed germination occurs only under these favorable conditions [7]. There were ≤ 3 days to first germination of most species in our study area (Figure 5). Such rapid germination could help ensure survival under unpredictable amounts and distribution of rain, because roots could quickly penetrate into the soil before the surface becomes dry [7]. This strategy ensures rapid seedling emergence and establishment after a very low amount of precipitation.

The proportion of species with seed dormancy in this area was 82.9%. However, C. C. Baskin and J. M. Baskin reported that 95% of the species in cold deserts are dormant at maturity [53]. Previous studies found different relationships between seed mass and germination in different ecosystems. The relationship between seed mass and germination was insignificant in tropical forests in Malaysia and Panama [54]. Chen et al. found a weak correlation between germination and seed mass in subtropical forests in China [55]. However, other researchers found a negative or significantly negative correlation between germination percentage and seed mass across species in subalpine forests on the Qinghai-Tibetan Plateau of China [56], in alpine meadow area in the Qinghai-Tibet Plateau of China [57], and in arid and semiarid zones in Inner Mongolia of China [50, 58]. Our results indicated that germination percentages of species with small seeds tended to be higher than those with large seeds, but the relationship was not statistically significant (Figure 6(a)). These results agree with the data collected in arid and semiarid zones [50, 58] and the differences might be related to the different environmental conditions in the various ecosystems. Small and rounded seeds germinated faster than large and flat ones in our study. This strategy would allow small seeds to colonize preoccupied suitable microsites in advance of larger-seeded, more competitive species [54]. Norden et al. suggested that the embryo of a large seed may require more time being grown within the seed and thus to germinate than the embryo in small seeds. In which case, large seeds would be expected to germinate more slowly than smaller ones [54].

There are many studies about the relationship between life form and seed germination strategy [53]. Garwood found no consistent correlation between life form and germination parameters in neotropical forest flora [59]. Wang et al. found that there was no relationship between life form and seed germination in arid and semiarid zones in China [50]. In contrast, Grime et al. found that seeds of annuals and perennial herbs germinated faster than those of shrubs and trees in a temperate flora [60]. Figueroa and Armesto found that seed germination of trees was significantly delayed relative to shrubs, vines, and herbs in Argentina [61]. Bu et al. proposed that the seeds of woody plants germinated earlier and to higher percentages than those of graminoids and forbs in the alpine meadow on the eastern Qinghai-Tibet Plateau [57]. We found that life form had a significant effect on final germination percentages in the Gurbantunggut Desert. And, to our surprise, the seeds of ephemeral plants and shrubs had higher germination percentages than those of annuals and perennial herbs (Figure 7).

Ephemeral plants are a special category in the Gurbantunggut Desert in that they depend on water from snowmelt to germinate, establish seedlings, and complete their life history in spring. As an adaptation to desert environments, ephemeral plants have fast and high germination when conditions are suitable. But why are germination percentages of shrubs higher than that of other life forms? Bu et al. gave a reasonable explanation: faster seed germination of shrubs might be due to their slower growth rate relative to the herbs. Thus, faster germination of shrubs would help them obtain competitive advantages in time and space [57].

The site where mother plants grow is identified as favorable by successful reproduction, and the best strategy would be to keep as many seeds as possible at the site. Ellner and Shmida studied species in the Negev Desert and suggested that the area near the mother plant was a “safe site” for seed germination and seedling survival [24]. In our study, species with ombrohydrochory had the highest germination percentages (Figure 6). The prolonged wet period enables mucilaginous seed to germinate even when they are situated on the soil surface [7]. Mucilage can aid in root penetration and in anchoring the seedling and by preventing collection of seeds by predators via adherence of seeds to the soil until they germinate [7]. Seeds of both anemochorous and zoochorous species potentially have a greater dispersal distance from the mother plant than seeds with ombrohydrochory and barochory [51]. Furthermore, some seeds with far distance dispersal (by wind or animals) germinated to a lower percentage than those dispersed a short distance, because sites far from the mother plants are unpredictable in the desert. Although autochorous species dispersed seeds near the mother plant, they germinated to the lowest percentage. The possible interpretation of this is that the autochorous species belong to Fabaceae, a family in which most seeds have an impermeable seed coat that prevents water uptake and thus seed germination [53].

Gutterman concluded that plants in the Negev Desert with seed protection strategies may develop cautious germination strategies, and plants with seed escape strategies may develop opportunistic germination strategies [7]. Our results showed that germination percentage did not differ in different dispersal strategies (Figure 7). Thus, Gutterman's conclusion does not apply to plants in the Gurbantunggut Desert. This demonstrates that plant species with the same dispersal strategies could have different germination strategies in different areas. Thus, seed dispersal strategies and seed germination strategies may be closely related to the environment in which a plant population occurs and then be influenced by different natural selection forces.

## Figures and Tables

**Table 1:** Family, species, vegetative period, month of seed collection, seed traits (seed mass, seed size, seed shape), seed germination percentage, dispersal syndromes and dispersal strategies recorded for each of the 70 study species.

Family	Species	Life form	Collecting time (months)	Seed mass (mg)	Seed size (mm)	Seed shape (variance)	Seed germination percentage (%)	Dispersal syndromes	Dispersal strategies
Ephedraceae	*Ephedra przewalskii *Stapf	LS	9	2.81 ± 0.071	3.926 ± 0.054	0.144	0	Anemochory	Escape-protection

Poaceae	*Eragrostis minor *Host.	E	8	0.076 ± 0.0024	0.584 ± 0.021	0.028	5.5 ± 2.22	Anemochory	escape
	*Eremopyrum triticeum* (Gaertn.) Nevski.	E	6	1.78 ± 0.046	11.389 ± 0.792	0.184	87.33 ± 2.40	Epizoochory	Protection
	*Leymus angustus* (Trin.) Pilger.	LPH	8	7.85 ± 0.68	9.534 ± 0.775	0.178	0.5 ± 0.5	Epizoochory	Protection
	*L. racemosus *(Lam.) Tzvel.	LP	8	9.44 ± 0.14	13.708 ± 0.872	0.156	47 ± 2.08	Epizoochory	Protection

Papaveraceae	*Glaucium squamigerum *Kar. et Kir.	LBP	6	0.42 ± 0.0018	1.269 ± 0.044	0.065	30 ± 5.29	Autochory	Escape

Ranunculaceae	*Ceratocephalus testiculatus *(Crantz) Bess.	E	6	1.06 ± 0.0099	4.614 ± 0.229	0.018	20.67 ± 1.76	Epizoochory	Escape-protection

Caryophyllaceae	*Minuartia regeliana *(Trautv.) Mattf.	LA	9	0.20 ± 0.0087	0.499 ± 0.011	0.028	0	Barochory	Escape

Amaranthaceae	*Agriophyllum squarrosum *(L.) Moq.	LA	9	1.16 ± 0.027	2.208 ± 0.093	0.108	8.5 ± 2.22	Barochory	Protection
	*Amaranthus albus* L.	LA	9	0.70 ± 0.0046	2.083 ± 0.058	0.069	14.5 ± 5.50	Barochory	Escape
	*A. retroflexus* L.	LA	10	0.68 ± 0.0088	2.115 ± 0.034	0.066	56 ± 2.00	Barochory	Escape
	*Anabasis aphylla* L.	LS	10	1.14 ± 0.020	2.064 ± 0.062	0.104	66.5 ± 4.03	Anemochory	Protection
	*Atriplex tatarica* L.	LA	10	1.10 ± 0.032	4.472 ± 0.355	0.168	11 ± 4.65	Anemochory	Escape-protection
	*Bassia dasyphylla* (Fisch. et Mey.) O. Kuntze.	LA	9	0.66 ± 0.0078	3.018 ± 0.145	0.15	15 ± 1.91	Epizoochory	Protection
	*B. hyssopifolia* (Pall.) O. Kuntze.	LA	10	0.43 ± 0.0049	2.958 ± 0.165	0.04	85.5 ± 4.27	Epizoochory	Protection
	*Camphorosma monspeliaca* L	LS	10	0.54 ± 0.015	2.238 ± 0.066	0.083	96 ± 5.48	Anemochory	Escape
	*Ceratocarpus arenarius* L.	LA	9	1.51 ± 0.025	7.624 ± 0.556	0.364	42.5 ± 2.36	Epizoochory	Escape-protection
	*Chenopodium album* L.	LA	7	0.35 ± 0.0017	0.964 ± 0.025	0.078	98.5 ± 0.96	Barochory	Escape
	*C. aristatum *L.	LA	8	0.11 ± 0.0022	0.594 ± 0.023	0.037	6.5 ± 0.96	Barochory	Escape
	*C. glaucum* L.	LA	9	0.22 ± 0.0021	0.864 ± 0.049	0.074	99 ± 0.58	Barochory	Escape
	*C. hybridum *L.	LA	10	0.35 ± 0.0016	1.776 ± 0.086	0.044	7.5 ± 2.22	Barochory	Escape
	*Corispermum lehmannianum *Bunge	LA	8	0.73 ± 0.01	2.634 ± 0.083	0.143	0	Epizoochory	Escape
	*Halogeton glomeratus *(Bieb.) C. A. Mey.	LA	10	1.06 ± 0.011	2.092 ± 0.047	0.088	77.00 ± 2.34	Anemochory	Escape-protection
	*Halostachys caspica *C. A. Mey. ex Schrenk.	LS	10	0.24 ± 0.0074	1.798 ± 0.077	0.046	69 ± 3.00	Anemochory	Escape
	*Haloxylon ammodendron *(C. A. Mey.) Bunge.	LS	10	3.89 ± 0.12	10.676 ± 0.419	0.172	73.5 ± 5.74	Anemochory	Protection
	*H. persicum* Bunge ex Boiss. et Buhse.	LS	10	7.97 ± 0.085	9.528 ± 0.149	0.147	54 ± 4.55	Anemochory	Protection
	*Horaninowia ulicina *Fisch. et Mey.	LA	9	0.23 ± 0.0057	0.932 ± 0.043	0.083	4 ± 1.41	Epizoochory	Escape
	*Kalidium caspicum* (L.) Ung.—Sternb.	LS	10	0.15 ± 0.002	1.568 ± 0.080	0.032	96.5 ± 1.71	Anemochory	Escape
	*K. foliatum* (Pall.) Moq.	LS	10	0.16 ± 0.0065	3.712 ± 0.213	0.047	98.00 ± 0.77	Anemochory	Escape
	*Kochia iranica* Litv. Ex Bornm.	LA	10	0.47 ± 0.008	0.698 ± 0.017	0.104	68 ± 2.45	Anemochory	Escape
	*Petrosimonia sibirica *(Pall.) Bunge.	LA	9	1.75 ± 0.015	0.728 ± 0.029	0.129	3 ± 1.29	Anemochory	Escape-protection
	*Salicornia europaea* L.	LA	10	0.055 ± 0.0004	1.270 ± 0.121	0.047	15 ± 3.87	Anemochory	Escape
	*Salsola foliosa* (L.) Schrad.	LA	10	1.04 ± 0.004	6.162 ± 0.218	0.182	0	Anemochory	Escape-protection
	*S. heptapotamica *Iljin.	LA	10	5.18 ± 0.069	10.176 ± 0.596	0.139	26 ± 8.21	Anemochory	Escape-protection
	*S. korshinskii *Drob.	LA	10	2.68 ± 0.053	8.312 ± 0.537	0.139	24 ± 9.42	Anemochory	Escape-protection
	*S. nitraria *Pall.	LA	10	1.80 ± 0.040	6.690 ± 0.269	0.115	43.5 ± 3.30	Anemochory	Escape-protection
	*S. ruthenica* Iljin.	LA	10	2.71 ± 0.041	8.216 ± 0.424	0.129	3 ± 1.29	Anemochory	Escape-protection
	*Suaeda acuminata *(C. A. Mey.) Moq.	LA	9	0.57 ± 0.017	1.463 ± 0.088	0.02	38.5 ± 1.89	Barochory	Escape
	*S. altissima* (L.) Pall.	LA	10	0.35 ± 0.0057	1.244 ± 0.033	0.029	24 ± 2.83	Barochory	Escape
	*S. microphylla *(C. A. Mey.) Pall.	LS	10	0.32 ± 0.0051	1.086 ± 0.040	0.003	52.5 ± 5.62	Barochory	Escape
	*S. physophora *Pall.	LS	10	2.48 ± 0.021	2.934 ± 0.162	0.029	79 ± 2.65	Anemochory	Escape-protection
	*S. salsa* (L.) Pall.	LA	10	0.15 ± 0.0028	0.792 ± 0.031	0.034	29 ± 1.91	Barochory	Escape

Polygonaceae	*Calligonum ebi-nurcum* Ivanova ex Soskov.	LS	8	26.72 ± 1.21	10.138 ± 0.369	0.045	1 ± 0.58	Anemochory	Escape-protection

Tamaricaceae	*Reaumuria songarica* (Pall.) Maxim.	LS	8	9.16 ± 0.12	6.050 ± 0.156	0.078	31.5 ± 2.50	Anemochory	Escape-protection

Plumbaginaceae	*Limonium coralloides *(Tausch) Lincz.	LP	8	0.26 ± 0.0063	2.622 ± 0.048	0.051	34.5 ± 12.53	Anemochory	Escape
	*L. suffruticosum *(L.) Kuntze.	LS	8	0.30 ± 0.0052	2.784 ± 0.157	0.109	29 ± 6.56	Anemochory	Escape

Fabaceae	*Alhagi sparsifolia* Shap.	LS	10	4.87 ± 0.064	3.672 ± 0.129	0.078	7.5 ± 1.89	Autochory	Escape-protection
	*Glycyrrhiza inflata* Batal	LP	8	37.90 ± 0.54	11.424 ± 0.639	0.091	8.23 ± 1.50	Autochory	Escape-protection
	*G. uralensis *Fisch.	LP	8	9.04 ± 0.060	2.796 ± 0.151	0.038	7.5 ± 0.96	Autochory	Escape-protection
	*Sophora alopecuroides* L.	LP	8	19.00 ± 0.19	3.968 ± 0.074	0.039	9.13 ± 1.67	Autochory	Escape-protection
	*Trigonella arcuata* C.A. Meyer	E	6	1.01 ± 0.018	2.248 ± 0.059	0.105	14.67 ± 1.33	Autochory	Escape-protection
	*T.cancellata* Desf.	E	6	0.88 ± 0.0096	2.100 ± 0.030	0.107	14.00 ± 3.06	Autochory	Escape

Zygophyllaceae	*Nitraria roborowskii *Kom.	LS	7	46.00 ± 0.91	7.916 ± 0.174	0.059	0	Endozoochory	Protection
	*Sarcozygium xanthoxylon* Bunge.	LS	8	106.53 ± 2.92	28.588 ± 1.133	0.051	83.5 ± 3.10	Anemochory	Escape-protection
	*Zygophyllum fabago* L.	LP	7	2.98 ± 0.92	3.704 ± 0.045	0.123	50.5 ± 6.70	Anemochory	Escape-protection

Brassicaceae	*Alyssum desertorum* Stapf.	E	6	0.37 ± 0.0050	1.474 ± 0.043	0.107	93.33 ± 1.76	Ombrohydrochory	Escape
	*Chorispora tenella* (Pall.) DC.	E	6	0.86 ± 0.0023	1.024 ± 0.040	0.08	6 ± 3.06	Ombrohydrochory	Escape
	*Descurainia sophia *(L.) Webb. ex Prantl.	E	6	0.11 ± 0.0007	0.916 ± 0.025	0.081	23.33 ± 2.40	Ombrohydrochory	Escape
	*Diptychocarpus strictus *(Fisch. ex. M. Bieb.) Trautv.	E	6	0.92 ± 0.0068	1.016 ± 0.038	0.074	46 ± 7.21	Ombrohydrochory	Escape
	*Euclidium syricum *(L.) R. Br.	E	6	4.01 ± 0.065	3.740 ± 0.163	0.066	3.33 ± 0.67	Epizoochory	Escape-protection
	*Neotorularia korolkowii *(Rgl. et Schmlh.)	E	6	0.095 ± 0.0011	0.966 ± 0.037	0.117	54.67 ± 8.74	Ombrohydrochory	Escape
	*Syrenia siliculosa* (M. Bieb.) Andrz.	E	6	0.21 ± 0.0039	1.588 ± 0.090	0.103	86.67 ± 7.33	Ombrohydrochory	Escape
	*Tauscheria lasiocarpa* Fisch. ex DC.	E	6	2.24 ± 0.018	3.038 ± 0.115	0.095	18.67 ± 5.70	Epizoochory	Escape-protection

Plantaginaceae	*Plantago cornuti *Gouan.	LP	9	0.55 ± 0.0055	1.244 ± 0.054	0.115	89.33 ± 0.67	Ombrohydrochory	Escape
	*P. lessingii *Fish. et Mey.	E	6	1.94 ± 0.021	3.546 ± 0.101	0.129	97.33 ± 0.67	Ombrohydrochory	Escape-protection
	*P. maritima *L. subsp. *ciliata* Printz.	LP	8	0.44 ± 0.0057	1.756 ± 0.054	0.105	93 ± 3.32	Ombrohydrochory	Escape
	*P. minuta* Pall.	E	6	2.04 ± 0.054	3.534 ± 0.090	0.129	76.67 ± 0.67	Ombrohydrochory	Escape-protection

Compositae	*Artemisia ordosica* Krasch.	LS	9	2.46 ± 0.040	1.546 ± 0.033	0.115	78.5 ± 3.20	Ombrohydrochory	Escape-protection
	*Cousinia affinis *Schrenk.	LP	8	4.25 ± 0.041	4.876 ± 0.196	0.093	14 ± 2.45	Epizoochory	Protection
	*Onopordum acanthium* L.	LB	8	9.82 ± 0.076	4.852 ± 0.053	0.09	82.5 ± 2.06	Barochory	Escape-protection

Notes: LA: long-lived Annuals; LBP: long-lived biennials-perennials; LPH: long-lived perennials; LS: long-lived shrubs; E: annuals/ephemerals.

**Table 2:** Dispersal syndromes and characteristics of diaspores and number of species, genera, and families with each syndrome.

Dispersal syndrome	Secondary dispersal syndrome	Fruit type of storage material	Fruit or seed trait relevant to dispersal	Number of species	Number of genera	Number of families
Zoochory	Endozoochory	Berry, drupe, storage material (sugars, starches, lipids, or proteins), or capsule	Edible aril or pulp	1	1	1
	Epizoochory	Hook-like or sticky substance capsule	Adherence structure	11	10	5

Anemochory		Capsule, pod, and winged nut; dust seed (<0.01 mg); hairy; and pappus	Easily dispersal by wind	25	17	7

Autochory		Explosive capsule	Ballistic	7	5	2

Barochory		None	Seed dispersal via gravity	15	8	4

Ombrohydrochory		Mucilage	Seed produces mucilage when wetted	11	8	3
